# Does rigid bracing provide the best outcome for children with neurological onset scoliosis?

**DOI:** 10.1186/1748-7161-8-S1-P9

**Published:** 2013-06-03

**Authors:** M Matthews

**Affiliations:** 1 DM Orthotics Ltd, Redruth, UK

## Background

Rigid bracing has been the mainstay of orthotic intervention for children with neurological onset scoliosis. The use of rigid braces, however, often do not provide the expected outcomes clinicians are hoping for with Cobb angles curve increasing over time [1], linked to worsening sitting ability and hip pain [2]. The time has come for a review of this particular type of scoliosis intervention.

## Aim

This paper will discuss the use of dynamic elastomeric fabric orthoses (DEFO) in this client group and question whether rigid bracing is most appropriate for long term outcomes. It will also discuss whether waiting for the 25 degree Cobb threshold, before treatment, is prudent.

## Methods

This study will use single case presentations (n=5) to demonstrate the advantages of early intervention using both radiographs and pre and post intervention photographs to illustrate the outcomes possible. All of the children have neurological onset scoliosis and show short/ medium term outcomes.

## Results

The use of DEFOs to control scoliosis has provided clinicians with an opportunity with which to reduce the number of children presenting for orthopaedic spinal surgery. Clinically the scoliosis clinic at the Norfolk & Norwich University Hospital has seen a declining number of children referred for scoliosis surgical management over the last ten years due to early intervention. The DEFO scoliosis suits appear to work by providing heightened proprioceptive input to the brain coupled with dynamic corrective forces to correct and re- align the spinal segments[3]. The DEFO suit provides a cosmetic orthosis capable of providing a continuous low level dynamic force capable of re-educating the brain to accept an improved posture. This process can be seen clinically over years resulting in re-learnt spinal symmetry and a reduced referral to the orthopaedic department. The use of rigid braces to control neurological onset scoliosis should now be reviewed by clinicians with a view to using more dynamic and cosmetic DEFO intervention options, which can reduce the need for surgery in the long term. Further larger scale research is required.
